# Nuclein

**Published:** 1895-08

**Authors:** E. Bergstretser


ARTICLE IV.

### NUCLEIN.

BY. DR. E. BERGSTRETSER.

The physiological process that presides over the nutrition of the body, that guards it from the invasions of a myriad of ever-active foes, and that is ever busy in restoring worn-out parts of the animal economy, is at the present time receiving a most searching analysis. Nutrition, prevention of disease, and cure are not now looked upon as distinct manifestations of vital energy, but are considered to be the phenomena of one ultimate force.

The cell has long been known to be the unit of vital action, but just in what way muscular, nervous or bone tissue were evolved from muscle, nerve or bone cells has been a trying question to physiologists. Surrounding the nucelus and the nucleolus of cells is found a granular substance called the blastema, and this substance becomes endowed by the cell with a certain molecular force that gives it activity in assimilation, even after it has become separated from its parent cell. This blastema is the substance that the nucleus and nucleolus of the cell use to produce and repair their own tissues. All cells possess this granular substance, or blastema, in varrying amounts, and it has been found broken off, or isolated, in the blood serum. Late investigators seem to have established the fact that this granular substance, found so universally throughout the animal economy, in the structure of its cells, and separate and free in the blood serum, is largely produced by the multinulear white blood-corpurcles. As these cells circulate through the blood-vessels and tissues they are secreting or manufacturing this granular substance which the individual tissue cells constantly seize upon, and by virtue of the selective activity of their nucleus and nucleolus, differentiate into their own peculiar tissue. In a crude way the multi-nuclear white blood-corpuscles may be compared with the numerous milk wagons of a large city, dirtributing to their thousands of hungar constituents that most nourishing fluid.

This granular substance, so largely produced by the multinuclear white blood-corpuscles, has lately received the name of "nucelin," although this same term has long been applied to the chemical basis of all animal cell structures. The later use of the word "nuclein" is both to designate the derivation of the substance so called and also indicate the relation that it sustains to the various tissue cells of the body, in furnishing them with the nutriment on which their structure and existence depend. The phenomena of nutrition consists of a gathering and of a separating process. First, the white blood corpuscles, when the products of digestion have entered the circulation, take hold of them, and, by an active dyalitic process, convert them into the granular substance called nuclein. Thus

Primary assimilation is accomplished by cellulation, and not in the crude manner described as absorption, or asmosis. Second, the individual tissue cells then take from the nuclein, found everywhere in the blood serum, just such parts of it as are adapted to the production of their own peculiar tissue. This is secondary assimilation or in reality separation.

The chemical symbol of nuclein is represented as $C_{27}$ $H_{49}$ $N_{9}$ $P_{3}$ O . It is freely soluble in alkaline solutions; slightly soluble in water, and is colorless. Nuclein belongs to the proteids, and contains a remarkably large amount of phosphorous, which exists in the form of nucleinic acid. The nucleinic acid of the various nucleins derived from differenc kinds of cells is always the same, but it is combined with varying basic substances in these different nucleins. These varying basic substances on decomposition yield up the xanthin bodies—*i. e.*. xanthin, adenin, guanin and sarkin. When nucleinic acid combines with a base which on decomposition yields adenin, it could be called adenin-nucleinic acid, and in the same way we could speak of guanin-nucleinic acid, or sarkin-nucleinic acid. Nuclein is isolated from the other proteids by nature of its strong resistance to the process of peptic digestion, under which the other proteids succumb. After these general statements as to the nature and origin of nuclein, we will now look into the method that nature takes to defend herself from the invasion of the destructive organisms of bacterial life.

A short summary of Prof. Vaughn's historical sketch of the search that physiologists have made for the germicidal agent of the blood, and his critical deductions, in so far as they bear on the line of thought presented in this paper, may prove of interest. In a series of experiments conducted by Louis and D. Cunningham, in 1872, it was found that babteria injected into the circulating blood rapidly disappeared. The earliest germicidal discoveries with extra vascular blood were made by Grohsmann, in 1884, who, in searching for the cause of coagulation of the blood, found that anthrax bacilli, after being kept in plasma, were less virulent when injected into the circulation of rabbits. In 1877 Fodor showed the germicidal action of the circulating blood on anthrax cultures, and also demonstrated that after a while this same blood lost its germicidal properties, the number of anthrax bacilli again increasing. In 1888 Nuttall found the same results as Fodor obtained held true in reference to the germicidal action of the blood of various animals on bacillus anthracis, bacillus subtillis, bacillus megaterium and staphyloccus pyogencs aureus. He used defibrinated blood in these experiments. Nissen, working under the direction of Flugge, as did also Nuttal, concluded from his experiments that:

"1. Cholera germs and Eberth's bacilli are easily destroyed by fresh blood.

"2. For a given amount of blood there is a maximum amount of bacilli that can be destroyed.

"3. Blood whose coagulability has been destroyed by the injection of pepsin is still germicidal.

"4. Filtered blood-plasma from the horse is germicidal."

Buchner, with the assistance of Voit, Sittman and Othenberger, in 1890, made the following demonstrations:

"1. The germicidal action of the blood is not due to phagocytes, because it is not influenced by the alternate freezing and thawing of the blood, by which the leucocytes of the rabbit are destroyed.

"2. The germicidal properties of the cell free serum must be due to its soluble constituents.

"3. Neither neutralization of the serum, nor the addition of pepsin, nor the removal of carbon-dioxide gas, nor treatment with oxygen has any effect on the germicidal properties of the blood.

"4. The inorganic salts have in or of themselves no germicidal action. They are active only in so far as they affect the normal properties of the albuminates of the serum. The germicidal properties of the serum reside in the albuminose constituents."

From this last conclusion of Buchner's, Prof. Victor Vaughn takes decidedly different views. For how, as he suggests, could serum-album remain in the blood-serum after the latter had undergone thorough peptic digestion. The search for the germicidal constituent of the blood has also been pursued by Halliburton, who called it a cell globulin, which he obtained from the lymphatic glands. Christmas found a germicidal substance in the spleen and other organs that he prepared in the form of a glycerine extract.

The proteids of the blood-serum, as containing the possible germicidal agent, attracted such investigators as Bitter, Christmas, Emmerich, Tsuboi, Steinmetz and Low, but they all concluded that serum-album was the germicidal agent of the blood. It is to the credit of American physiologists that Prof. Victor Vaughn, of the University of Michigan, was the first to demonstrate ultimately the germicidal agent of the blood. He has shown by a large number of experiments that consisted of a complete process of peptic digestion of the albuminose constituents of the blood serum that:

"1. Serum-album is not the germicidal agent of the blood.

"2. The germicidal substance must belong to the proteids; otherwise it would be difficult to explain the fact that a temperature of 556 renders blood serum inactive.

"3. The only proteid likely to be present in blood-serum and which is not destroyed by peptic digestion is *nuclein.*"

A detailed description of Prof. Vaughn's method of obtaining nuclein from blood-serum, which he was the first to do, would not be in a direct line at this time. It is only necessary to state that he used the blood-serum from rabbits and dogs. The whole process was, of course, conducted with perfect antisepsis, and was in its general details a thorough peptic digestion of the proteids of the blood-serum that could be affected in that manner, thus leaving isolated the nuclein.

The following series of experiments demonstrate Vaughn's conclusions quoted above:

*Experiment I.*—A nuclein tube was inoculated with the bacillus of *Asiatic Cholera*, and plates made from this gave the following results:

Time: Immediately. 5 min. 15 min. 30 min. 1 hr. 1½ hr. 22 hrs.

No. of Colonies: 2100 43 54 71 90 115 1200

That the alkali in which this nuclein was dissolved did not cause the decrease in the number of germs is shown by the subsequent increase.

*Experiment II.—Staphylococcus Pyogenes Aureus.*

| Time: | Immediately. | 1 hour. | 4 hours. | 7 hours. | 24 hours. |
|---|---|---|---|---|---|
| No. of Colonies: | 4000 | 1720 | 1050 | 810 | 0 |

*Experiment III.—Anthrax Bacillus without spores.*

| Time: | Immediately. | 1 honr. | 4 hours. | 7 hours. | 24 hrs. |
|---|---|---|---|---|---|
| No. of Colonies: | 100 | 43 | 10 | 1 | 0 |

*Experiment IV.—Cholera Germ.*

| Time: | Immediately. | 1 hour. | 4. hrs. | 7 hrs. | 24 hrs. |
|---|---|---|---|---|---|
| No. of Colonies: | 470 | 45 | 1 | 0 | 410 |

The final increase in the number of cholera germs occurred both in the nuclein solution prepared from the serum of the rabbit and that prepared from the serum of the dog.

*Experiment V.—Staphylococcus Pyogenes Aureus.*

| Time: | Immediately. | 1 hour. | 5 hrs. | 19 hrs. | 24 hrs. |
|---|---|---|---|---|---|
| No. of Colonies: | Countless. | 22,000 | 12,525 | 155 | 0 |

*Experiment VI.—Anthrax Bacillus without Spores.*

| Time: | Immediately. | 1 hour. | 5 hrs. | 19 hrs. | 24 hrs. |
|---|---|---|---|---|---|
| No. of Colonies: | 5000 | 165 | 0 | 0 | 0 |

*Experiment VII.—Staphylococcus Pyogenes Aureus.*

| Time: | Immediately. | 1 hour. | 4 hrs. | 7 hrs. | 24 hrs. |
|---|---|---|---|---|---|
| No. of Colonies: | 5000 | 2500 | 1600 | 1200 | 0 |

*Experiment VIII.—Anthrax Bacillus without Spores.*

| Time: | Immediately. | 1 hour. | 4 hrs. | 7 hrs. | 24 hrs. |
|---|---|---|---|---|---|
| No. of Colonies: | 43 | 7 | 0 | 0 | 0 |

*Experiment IX.—Cholera Bacillus.*

| Time: | Immediately. | 1 hour. | 4 hrs. | 7 hrs. | 24 hrs. |
|---|---|---|---|---|---|
| No. of Colonies: | 350 | 105 | 150 | 42 | 0 |

*Experiment X.—Staphylococcus Pyogenes Aureus.*

| Time: | Immediately. | 1 hour. | 5 hrs. | 19 hrs. | 24 hrs. |
|---|---|---|---|---|---|
| No. of Colonies: | Countless, | 25,000 | 5525 | 65 | 500 |

*Experiment XI.—Anthrax Bacillus withont Spores.*

| Time: | Immediately. | 1 hour. | 5 hrs. | 19 hrs. | 24 hrs. |
|---|---|---|---|---|---|
| No. of Colonies: | 430 | 0 | 0 | 0 | 0 |

The volume of the solvent used to dissolve the nuclein in these cases was the same as the blood-serum from which the nuclein was obtained. Sterilized water was used as the solvent and it contained 0.12 per cent of potassic hydrate and 0.06 per cent of sodium chloride. In the last five experiments the sterilized water contained 1.2 grams of potassic hydrate, 6 grams of sodium chloride, and 1 gram each of sodium bicarbonate and disodium hydrogen phosphate to one liter of water.

These experiments are so absolutely conclusive, and of so great interest and importance to the dentist in the treatment of the pathological conditions that he finds in his work, that I have brought them into this paper. So far as I am aware, dental literature has not contained any reference to them.

When infection of the tissue takes place, at once the leucocytes rush to the place where the poisonous germs are at work. The nuclein is poured out all around the invaded tissues, and the retrograde metamorphosis is checked and repaired. But, if the disease germ is one of strong vitality and rapid growth, the struggle becomes most intense, and may end in defeat for the leucocytes, for the thyrode and thymus glands, the spleen, and red marrow of the bones, from which the multi-nuclear white blood-corpuscles are now thought to originate, can respond to only a certain extent, and when they become exhausted the field is left to the triumphant bacteria and their ptomaines.

Just in what way the nuclein of the leucocyte overcomes or destroys the nuclein of the bacterial cell is a difficult problem to solve. Vaughn suggests the manner in which this may be brought about: The nucleins of the multi-nuclear white blood-corpuscles are isomeric bodies, one being designated as the A compound and the other as the B compound, and one, by causing a rearrangement of the atoms of the molecule of the other, may absorb it. The molecule with the most vitality will rearrange and absorb the molecule of less vitality. In this way we can understand how disease germs at sometimes are victorious and at other times are conquered in their attacks upon the germicidal cells of the blood. That vital force plays a great part in the drama is demonstrated by the fact that anthrax germs flourish in the extra-vascular blood of the chick while that fowl is from birth immune to their influence, and the extra-vascular blood of the rabbit destroys these same germs, which prove rapidly destructive to the animal when introduced into its circulation.

In many animals we find the condition of *natural* immunity to certain disease germs. The chicken from birth immune to anthrax bacillus, but in most cases of natural immunity to certain disease germs the condition is not perfected until development of the body is matured. The young rat is susceptible to anthrax bacillus, while the mature rat is immune. The human adult is almost perfectly immune to many of the infectious diseases of childhood. We also have examples of racial immunity to certain disease germs, the negro being largely exempt from malarial diseases.

*Artificial* immunity to disease germs is induced by the introduction of germ nuclein into the body. By this means the organs that produce the multi nuclear white blood-corpuscles are stimulated to increased functional activity and a supply of nuclein is furnished which eradicates the germs. As we all know, this immunity is liable to fade out. Vaccination loses its protective effect, and a second attack of scarlet fever, diphtheria or measles is not an uncommon occurrence.

Conditions of health have a very important bearing as to an individual's ability to resist infection. Exhausted conditions of the system may result in allowing the entrance of infectious germs, because Nature is not at once able to rally her protective and nutrition-bearing white blood-corpucles to the scene of invasion. If a rat be exhausted by prolonged exertion on a tread-mill, he readily succumbs to innoculation from anthrax germs, although in normal condition he is immune to them. Climatic cures are not so much the result of removal from unhealthy environment as a stimulation to the nuclein producing-organisms.

We are greatly indebted to Metschnikoff for his theory of phagocytosis, but he failed to distinguish the fact that it was not the cell itself, but its product, nuclein, that was the germicide, and that it is probably most active after it has separated from the parent cell and passed into the circulation. The blood-serum of rabbits is germicidal, even after alternate freezing and thawing of the leucocytes, which is destructive to them. Spores inclosed in bits of parchment and introduced under the skin of rabbits were destroyed. This result was accomplished under conditions where phagocytes were unable to enter because of the parchment. This experiment shows that the germicide must be a soluble constituent of the blood. Against Metschnikoff's theory is also arrayed the fact that germs grow and multiply in the phagocytes themselves, and in this way the latter may become carriers of infection.

There is an intimate connection between the formation of uric acid and nuclein which directly interests us as dentists in study of the ætiology of pyorrhœa alveolaris. Herbaczewski has recently added some important discoveries as to the origin of uric acid. Heretofore the formation of uric acid has been accredited to deficient oxidation of those materials the completed product of which is urea, but in physiology it has been difficult to demonstrate this supposed relation. Herbaczewski has shown that the nuclein of spleen pulp produces uric acid, in the presence of an oxidizing agent like the blood. The uric acid obtained by oxidizing spleen nuclein is not a ready-formed substance of the nuclein, but is the derivation of a mother substance that also produces xanthin, sarkin, guanin and adenin. This mother substance, found in all nucleins, when split up in the presence of an oxidizing agent, produces uric acid, while xanthin, sarkin, guanin and adenin are formed when disintegration of this mother substance takes place in the absence of an oxidizing agent. This is the most satisfactory explanation yet produced as to the formation of uric acid in the animal economy. The formation of uric acid and the xanthan bodied is the measure of the amount of nuclein being consumed, which primarily means the number of white blood-corpuscles undergoing destructive metamorphosis. Excessive uric acid formation then means abnormal increase of the white blood-corpuscles or leucocytosis. When nutrition is very active, yet normal, as in the infant, we find a large proportion of uric acid; and when nutrition is running low, as in stages of fasting, there is a diminished amount of uric acid. Those diseases that disarrange and cause to run wild the nutritive processes are accompanied by excessive uric acid and xanthan formations, and we now understand, through Herbaczewski's demonstrations, that this is caused by excessive formation and destruction of the white blood-corpuscles, as they no doubt furnish the largest part of the nuclein of the blood.

If the uric acid diathesis be the ultimate cause of pyorrhœa alveolaris, the dentist who attempts to cure it by local treatment only exhausts himself in a fruitless endeavor. Therapeutics are on the verge of a great transformation. It being known that nuclein is an antitoxine and a tissue builder, the great problem is now to obtain nuclein so as to preserve its functional activity. The stomatologist cannot but feel an interest in these new discoveries and applications of cure to disease. Our knowledge of the manner in which Nature keeps the animal economy in repair, and defends herself from the invasion of her bacterial foes has been revised and enlarged. In the discovery of the functional importance of nuclein and its manner of production, a new realm opens up to the physiologist and the therapeutist. The spleen, the hepatic cells, the thyroid and the thymus glands and testes have been found to be sources of this most important factor in the problem of life, but it is highly improbable that so vital an element should be confined alone to these corners of the animal economy. It probably will yet be proven that nuclein finds its sources from all the nucleated cells of the body.

In the treatment of many diseases that come under his care the dentist has frequently overlooked the fact that Nature has a most efficient germicide, soluble and everywhere present in the blood-serum. The appalling list of escharotics and other powerful medicaments that are used in the treatment of pyorrhœa alveolaris under these circumstances appears often unnecessary after thorough surgical removal of the exciting cause. It is utterly impossible for the oral surgeon to perform a perfectly aseptic operation from the very nature of the local environment. Yet no other class of surgical cases heal so kindly. The blood supply of the face and head is so abundant that Nature seems to be able with her own germicidal agent, nuclein, to do far more than the best aseptic surgery can accomplish in any other part of the body. This should be a strong hint to all of us. If more dependence were put on cleanliness and thorough surgery, and less on powerful and irritating germicides, Nature, through her own superior means, would show us results that are now seldom attained.—Proceedings of Kansas State Dental Association.—*Western Dental Journel.*

